# Photodynamics of Bright Subnanosecond Emission from Pure Single-Photon Sources in Hexagonal Boron Nitride

**DOI:** 10.3390/nano12244495

**Published:** 2022-12-19

**Authors:** Alexander V. Gritsienko, Aliaksandr Duleba, Mikhail V. Pugachev, Nikita S. Kurochkin, Igor I. Vlasov, Alexei G. Vitukhnovsky, Alexandr Yu. Kuntsevich

**Affiliations:** 1 P. N. Lebedev Physical Institute of the Russian Academy of Sciences, 53 Leninskiy Pr., 119991 Moscow, Russia; 2 Moscow Institute of Physics and Technology, National Research University, 9 Institutskií Per., 141700 Dolgoprudnyí, Russia; 3 Prokhorov General Physics Institute of the Russian Academy of Sciences, Vavilov str. 38, 119991 Moscow, Russia

**Keywords:** single-photon sources, hexagonal boron nitride, Hanbury Brown and Twiss interferometry, photobleaching, photodynamics

## Abstract

Bright and stable emitters of single indistinguishable photons are crucial for quantum technologies. The origin of the promising bright emitters recently observed in hexagonal boron nitride (hBN) still remains unclear. This study reports pure single-photon sources in multi-layered hBN at room temperature that demonstrate high emission rates. The quantum emitters are introduced with argon beam treatment and air annealing of mechanically exfoliated hBN flakes with thicknesses of 5–100 nm. Spectral and time-resolved measurements reveal the emitters have more than 1 GHz of excited-to-ground state transition rate. The observed photoswitching between dark and bright states indicates the strong sensitivity of the emitter to the electrostatic environment and the importance of the indirect excitation for the photodynamics.

## 1. Introduction

The development of on-demand single-photon emitters (SPEs) is crucial for applications such as quantum key distribution, optical quantum computing, radiometry, metrology, and others [1,2,3,4,5,6]. The most important features for the SPE practical applicability are purity, brightness (>1 MHz), photon emission rate (>1 GHz), and room temperature operation [7,8]. Recently, fluorescent defects in layered hexagonal boron nitride (hBN) have been explored as SPEs with stable ultrahigh brightness at room temperature, as well as high internal and external quantum yields [9,10,11,12,13]. Significant progress has been made in fabricating defects in hBN flakes. This includes plasma treatment [14], ion implantation [15], or deterministic fabrication using a probe [16], femtosecond pulses [17] or focused ions [18,19], and electron beams [20]. However, there is still no consensus about their photophysical properties, energy levels, and atomic structures [21,22,23,24], especially for the SPEs emitting within the most useful range of 1.9–2.2 eV.

Despite the high-brightness emission of SPEs in hBN, their emission rates, as a rule, do not exceed 1 GHz when special resonators are not involved and thus cannot be high enough for real applications [25,26,27,28,29,30]. Moreover, the properties of the investigated color centers may differ and have various photodynamic properties. That is, difficulties arise associated with the reproducibility of the centers made by different methods. Nowadays, it remains an important task to search for optimal methods for creating stable color centers that satisfy the previously specified criteria, including the possibility of optical or electrical control [31,32].

We report the fabrication and optical studies of the emitters in hBN flakes with a subnanosecond emission decay time. It has been shown that bright emitters satisfy most of the important criteria for quantum applications, such as high purity and a decay rate of more than 1 GHz in the zero-phonon line (ZPL) even at zero pumping power. The emitters demonstrate unusual photodynamics of switching between dark and bright states, which may include complete deactivation. The dynamics show the presence of an intermediate excited level close to the conduction band and indicate that the possible electron escape to neighboring defects can be the main deactivation mechanism.

## 2. Materials and Methods

**Figure 1:**
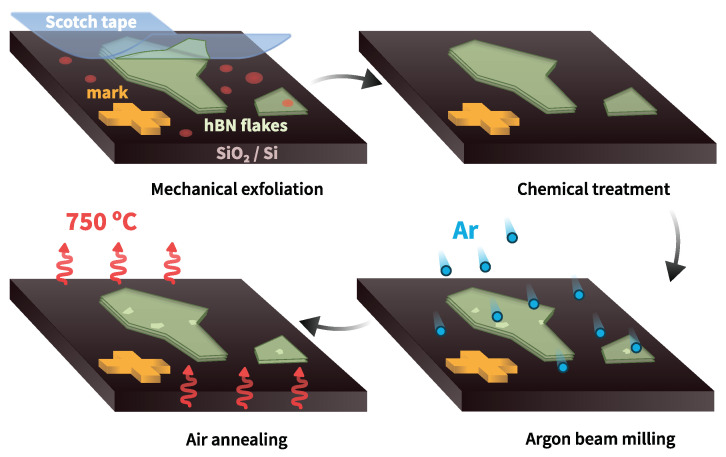
Sample fabrication scheme: mechanical exfoliation and transferring of hBN flakes to the substrate using scotch tape; chemical treatment to clean off the dirt and glue; introduction of structural defects in the flakes with an argon beam; high-temperature annealing in air to activate the emitters.

In our experiments, hBN flakes with thicknesses of about 5–100 nm were exfoliated from a commercially available hBN crystal using an adhesive tape and transferred to a pre-cleaned Si wafer with a 285-nm SiO${}_{2}$ layer [33] and lithographically patterned gold markers on the surface [34]. After the transfer, the wafer with the flakes was cleaned with acetone, isopropanol, and deionized water, and subsequently, hBN flakes were treated with argon atoms in the Plassys 550s setup to introduce structural defects in the hBN lattice. Argon beam milling was applied for 30 s at a 500 V beam voltage and a 100 V acceleration voltage (31.2 mAmps emission current). Thus, the concentration of structural defects on the surface of the hBN flakes studied in this work can be increased. Finally, to treat the residues of dirt and activate the emitters in the hBN flakes, we annealed the sample for an hour in air at 750 ${}^{{}^{\circ}}$C [35]. The main fabrication steps are shown in Figure 1.

**Figure A1:**
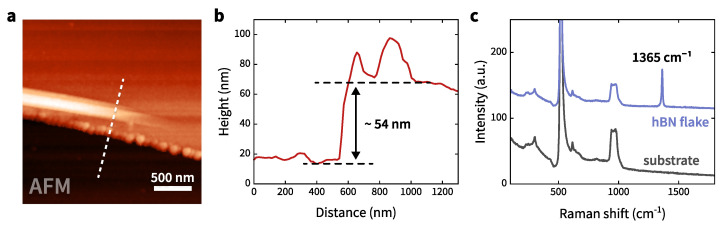
(**a**) AFM image of the hBN flake region and height profile (**b**) of the marked line in (**a**); (**c**) Raman spectra of the hBN flake on a silicon substrate.

The atomic force microscopy (AFM) (Solver Pro M, NT-MDT) was employed to study the sample with the hBN flakes. Measurements were carried out in a semicontact scanning mode. Raman spectroscopy was used to identify the hBN crystalline structure after treatment procedures, revealing the characteristic hBN E${}_{2g}$ Raman mode at 1365 cm${}^{-1}$ as shown in Figure A1c (see Appendix A).

## 3. Results and Discussions

### 3.1. SPE Identification

**Figure 2:**
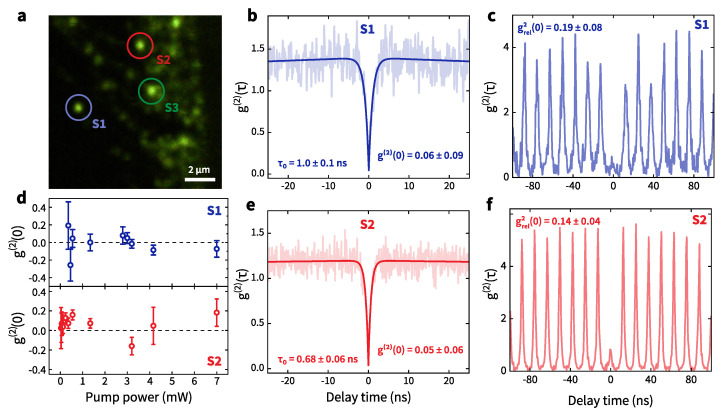
(**a**) Photoluminescence scanning image obtained under 532-nm CW laser illumination. Circles indicate quantum emitters. Autocorrelation functions $g^{\left(2\right)}\left(\tau \right)$ for the emitters S1 (**b**,**c**) and S2 (**e**,**f**) circled in (**a**) at ZPL emission under 532-nm CW and pulsed (80 MHz) 545-nm laser excitation. The absolute $g^{\left(2\right)}\left(0\right)$ values are given with IRF correction. Solid curves are a model fit as discussed in the text; (**d**) dependencies of extracted corrected $g^{\left(2\right)}\left(0\right)$ values for S1 (top) and S2 (bottom) on pump power at 532-nm excitation.

We studied micrometer-sized multilayer hBN flakes in a laser-scanning confocal microscope under continuous wave (CW) or pulsed excitation at 532 nm as described elsewhere [36,37]. It was found that individual flakes contained from several to dozens of quantum emitters located at a distance from the flake boundaries (more than 1–2 microns). Figure 2a demonstrates the flake region with a lot of bright emitters. We focus on S1 and S2 emitters circled in Figure 2a. The average thickness of the considered flake is of about 50 nm as shown in Figure A1a,b. Apparently, these defects are created by Ar beam treatment near the upper surface of the hBN flake according to previous studies [24].

We performed photon correlation measurements to obtain information about the photon statistics (Figure 2b,e) using a Hanbury Brown–Twiss (HBT) interferometer. To reduce the undesired emission in the photon statistics measurements, the spectral window was restricted to the ZPL region using narrow bandpass filters with a 20 nm transmission range. Note that there was no background correction. We extracted the essential parameters of quantum emission using the equation [38,39]:

(1) $$g^{\left(2\right)}\left(\tau \right)=1-C_{0}e^{-\left|\tau \right|/\tau_{0}}+C_{1}e^{-\left|\tau \right|/\tau_{1}},$$

where $\tau_{0}$ and $\tau_{1}$ are the decay times corresponding to excited and non-radiative metastable states, and $C_{0}$ and $C_{1}$ are antibunching and bunching amplitudes. For more accurate estimation of the $g^{\left(2\right)}\left(0\right)$ values, we took into account the instrument response function (IRF) for our setup in autocorrelation measurements as a Lorentzian function with ∼150 ps full width at half maximum (FWHM). As seen in Figure 2d, the designated emitters show pure single-photon emission even at high excitation power with $g^{\left(2\right)}\left(0\right)$ values close to zero. The $g^{\left(2\right)}\left(\tau \right)$ curves obtained at pulsed laser excitation demonstrate significant depletion of the $g^{\left(2\right)}\left(\tau =0\right)$ value (Figure 2c,f). Therefore, the presented sources can be used as on-demand quantum emitters.

**Figure A2:**
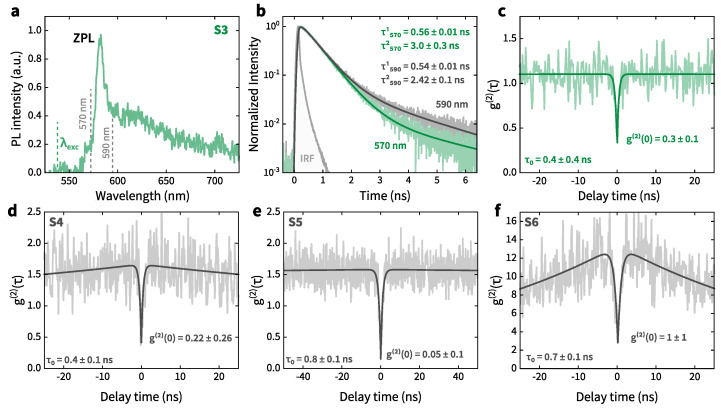
(**a**) The μ-PL spectra of the S3 emitter circled in Figure 2a under 532-nm excitation. The centers of the spectral regions of interest are indicated by dashed lines and have a bandwidth of 20 nm; (**b**) spectrally filtered PL decay curves and fits to the data for S3 at pulsed (80 MHz) 530-nm laser excitation. The light grey curve represents the IRF. Extracted decay times (τ) for each curve are presented on the top of the figure; (**c**–**f**) autocorrelation functions $g^{\left(2\right)}\left(\tau \right)$ for S3 (**c**) and other emitters S4–S6. The absolute $g^{\left(2\right)}\left(0\right)$ values are given with IRF correction.

The obtained characteristic decay times (e.g., 650 ps for S2, or 500 ps for S3, Figure A2) are not typical of hBN color centers studied previously (0.9–5 ns) [40,41,42,43]. To the best of our knowledge, so far, decay times shorter than 0.9 ns have been observed only by Ping Koy Lam’s group [24,44] for emitters in hBN flakes treated with oxygen plasma. However, in contrast to our study, the majority of these emitters were located on the flake boundaries, and their individual photophysical properties were not studied in detail. Moreover, a PL decay curve by itself does not provide sufficient information. This is due to the fact that the measurement of a decay time may depend on the excitation power due to the different absorption cross sections of individual emitters and different electron relaxation mechanisms [45]. Therefore, we provide appropriate optical measurements that can help describe the photodynamics in the hBN emitters. The corresponding results are discussed below.

**Figure 3:**
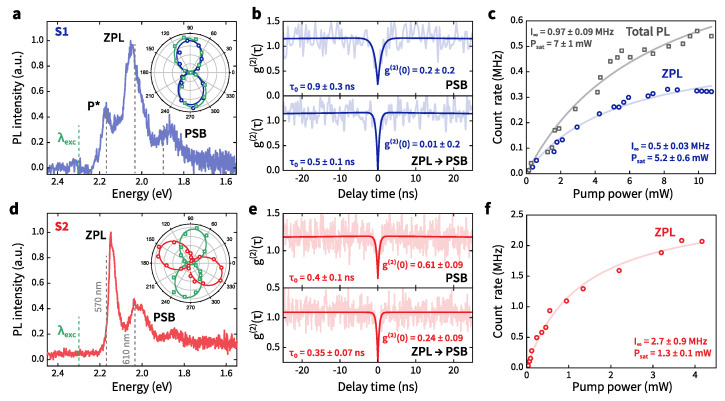
The μ-PL spectra of the emitters S1 (**a**) and S2 (**d**) under 532-nm excitation; the inset: PL intensity as a function of linear 532-nm excitation polarization angle (green hollow squares) or filtered by linear polarization angle in emission (blue and red hollow circles) for S1 and S2 emitters. Solid curves are fits to the data using the model discussed in the text. The photon autocorrelation of PSB (top) and cross-correlations between ZPL and PSB (bottom) for the emitters S1 (**b**) and S2 (**e**). The centers of the spectral regions of interest are indicated in (**a**,**d**) by dashed lines and have a bandwidth of 20 nm for the ZPL and PSB. Saturation curves for the S1 (**c**) and S2 (**f**) emitters; solid curves are fits to the data using an empirical model discussed in the text.

**Figure A3:**
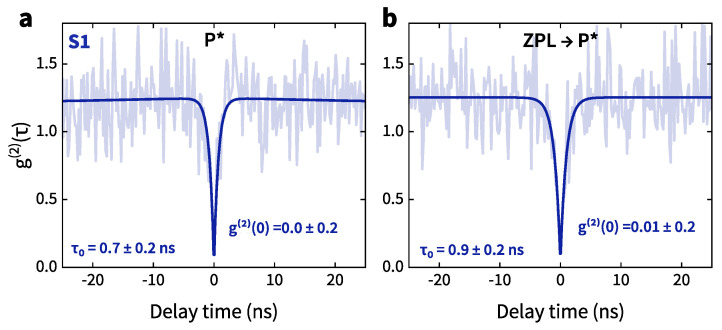
Autocorrelation functions $g^{\left(2\right)}\left(\tau \right)$ for S1 in the P*-line spectral range (**a**) and two-color cross-correlation between photons from P* and ZPL (**b**).

Figure 3a,d show the microphotoluminescence (μ-PL) spectra of S1 and S2 with the typical zero-phonon line and red-shifted phonon sideband (PSB). The origin of emission at 570 nm for S1 (satellite line, labeled as P*) is not clear. Such spectra have been previously observed for emitters in hBN, but their origins have not been considered in detail [10,42]. For better understanding, we try to identify a correlation between PSBs, the P* line, and the ZPL emission of the same emitter. For this, two-color HBT measurements were performed [46] as demonstrated in Figure 3b,e. The two-color correlation function $g_{l\to m}^{\left(2\right)}\left(\tau \right)$ quantifies the correlation between a photon of color **l** and a photon of color **m** detected at time τ later. Using the Cauchy–Schwarz inequality ${\left[g_{l\to m}^{\left(2\right)}\left(0\right)\right]}^{2}\leq g_{l}^{\left(2\right)}\left(0\right)\cdot g_{m}^{\left(2\right)}\left(0\right)$ [46], we conclude that the PSB line and the P* emission peak at 570 nm correspond to the same S1 emitter (Figure 3b and Figure A3, see Appendix C). A possible explanation is that the lines at 570 nm and 605 nm are two different ZPLs of the same emitter [47]. For convenience, by ‘ZPL’ we mean the most intense peak of S1. In the case of S2, PL near the PSB contains the emission from the extra emitter. As seen in Figure 3e (top figure), $g^{\left(2\right)}\left(\tau \right)$ does not satisfy the standard threshold $g^{\left(2\right)}\left(0\right)$ < 0.5 [38].

The inset to each PL spectra (Figure 3a,d) presents measurements of the polarized excitation and emission properties for the emitters. These data are acquired by either varying the linear polarization of the excitation laser (green hollow squares) or by passing the PL through a linear polarizer placed in the collection path (blue or red hollow circles). Solid curves are fitted to the data using the model function $I\left(\theta \right)=A\;cos^{2}\left(\theta -\theta_{0}\right)+B$, where θ corresponds to either the excitation or emission polarization angle; $\theta_{0}$ is the angle shift. *A* is the amplitude, and *B* is the offset. For S2, the excitation polarization dependence differs from the emission polarization pattern. This fact indicates indirect excitation of the defect [48].

The PL measurements with background corrections in Figure 3c,f show saturation count rates and saturation powers for the S1 and S2 emitters. The solid lines are fitted as $I=I_{\infty }\times P/\left(P+P_{sat}\right)$, where $I_{\infty }$ is the maximum count rate for P→∞, and $P_{sat}$ is the saturation power when $I=I_{\infty }/2$. The saturation measurements were taken by integrating the emission intensity over the ZPL through the corresponding bandpass filters. For S1, we also measured saturation curves (the total PL) with the P* and PSB emission taken into account. The considered emitters demonstrate the saturation count rates ≥ 0.5–2 MHz at the ZPL.

**Figure 4:**
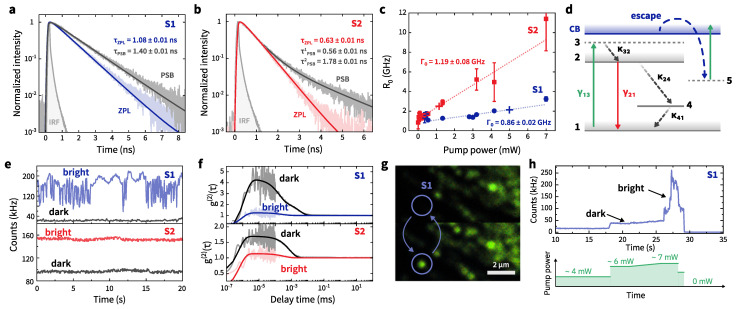
Spectrally filtered PL decay curves and fits to the data for S1 (**a**) and S2 (**b**) emitters at pulsed (80 MHz) 530-nm laser excitation. The light grey curve represents the IRF; (**c**) dependencies of transition rates for S1 and S2 on excitation power extracted from the $g^{\left(2\right)}\left(\tau \right)$ fit. The error bars represent one standard deviation. The cross mark indicates the saturation power for each emitter; (**d**) proposed energy level structure of an hBN defect with a metastable state indicated as 4; (**e**) PL trace for the S1 (top) and S2 (bottom) emitters at 532-nm laser excitation in the dark and bright states; (**f**) full-scale $g^{\left(2\right)}\left(\tau \right)$ functions for S1 (top) and S2 (bottom) in the dark and bright states. Solid curves are the $g^{\left(2\right)}\left(\tau \right)$ functions simulated to fit the experimental data; the procedure is described in Appendix B; (**g**) photoluminescence intensity map with deactivated (top) and activated (bottom) S1 emitter; (**h**) switching from the dark to the bright state for S1 emitter (top) via experimental protocol with 532-nm excitation (bottom).

To accurately estimate the excited-state decay time in quantum emitters S1 and S2, we perform (i) time-correlated single photon counting (TCSPC) measurements with pulsed excitation, and (ii) measurement of the photon correlation function to extract the decay time $\tau_{0}$ with different CW excitation power. The PL decay curves and their fits (Figure 4a,b) reveal single-exponential decays at the ZPL. It is noteworthy that the decay time at the PSB for S1 differs by 30% from the ZPL decay times. This partially explains why the pattern of two-color correlations for ZPL→PSB shows significant variation between the ZPL and PSB autocorrelation measurements. For S2, the PL decay for the PSB reveals a bi-exponential behavior with the contribution of the second exponential decay possibly corresponding to the second emitter as discussed above.

We observed the linear dependence of transition rates ($R_{0}=\;1/\tau_{0}$) on the excitation power for S1 and S2 at the ZPL as demonstrated in Figure 4c. The values for Figure 4c were obtained by extracting $\tau_{0}$ from $g^{\left(2\right)}\left(\tau \right)$ according to Equation (1). The data were fitted using the equation $R_{0}\left(P\right)=\Gamma_{0}+\sigma P$, where $\Gamma_{0}$ is the ZPL transition rate at zero excitation power, *P* is the excitation power, and σ is the fitting coefficient. The σ value reflects the emitter absorption cross section for a given wavelength and excitation polarization. Here, we ignored the presence of non-radiative relaxation from the excited to the ground states through a metastable state (Figure 4d). Decay times corresponding to the metastable states are 2–3 orders of magnitude longer than the excited-state decay times. According to Figure 4c, at P→0, the S2 emitter has $\Gamma_{0}$ that exceeds 1 GHz. In other words, the excited-state lifetime is less than 1 ns (∼840 ps).

### 3.2. Photoswitching

Some SPEs reveal blinking and photobleaching which indicate the presence of non-radiative relaxation channels for electrons in the defects. These channels represent the limitations standing in the way of achieving the high-rate and high-intensity emission important to real applications. For example, the S1 emitter can be deactivated (with no emission) by irradiation at high excitation power (>7 mW) for an arbitrarily long time (∼month) as shown in Figure 4g. If large excitation power is applied again, the emitter turns to its active state. However, after several such cycles of switching, the S1 emitter started to blink (Figure 4e). Let us call the state with low PL (incomplete deactivation) as dark, while the high-intensity state of the S1 emitter will be referred to as bright. The example of switching from the dark to the bright state for S1 is shown in Figure 4h. We found a significant increase in the measured $g^{\left(2\right)}\left(\tau \right)$ function (>4) for S1 in the dark state (Figure 4f) near τ=0. The S2 emitter also revealed a transition to a dark state with a PL intensity 1.5 times less than that of the bright state under high-power laser irradiation (>4 mW). Similarly to S1, the S2 emitter can recover the bright state after high-power laser irradiation. Figure 4f demonstrates the difference between the measured $g^{\left(2\right)}\left(\tau \right)$ for S2 in the bright and dark states. Note that the transition from the bright to the dark state for S1 or S2 under very high irradiation power (>7 mW) occurs randomly. At the same time, if they are not affected by high-power radiation, the emitters behave stably, i.e., they can remain in their bright or dark state for a long time (>1 day). All the main measurements above were carried out for emitters in these stable states.

For the scheme illustrated in Figure 4d and adopted from refs. [36,45,48], we used the simulation to solve the rate equation (see Appendix D) and to fit the experimental data in Figure 4f. The increase in the $g^{\left(2\right)}\left(\tau \right)$ values measured for the dark state results from a significant increase in the rate of electron transition to the metastable state ($\kappa_{24}$), whereas the rate of electron transition from the metastable to the lower state ($\kappa_{41}$) is obviously less (Table 1). The $g^{\left(2\right)}\left(\tau \right)$ function increases; hence, the total emission intensity decreases. Based on the previous studies [9,49], we believe that this is directly related to the atomic structure of the defect and its local stress or electrostatic environment.

Next, we will discuss the mechanisms for the photoswitching of the defects. The transition to a dark state or complete optical deactivation of the defect is associated with the photoionization or modification of the defect itself or the defects nearby [36,47,50]. Consider the example of the energy structure with indirect excitation of a defect. When a photon is absorbed, the electron is transferred from the lower (1, ground) to the upper excited state (3). If level (3) is close in energy to the conduction band (CB), there is a probability of electron capture by the CB. This is possible at high-power excitation due to, for example, an increase in the local heating of the crystal lattice and mutual displacement of energy levels. Then, an electron captured by the CB can escape to a neighboring defect (5, electron trap), while this defect changes its charge and loses the ability to re-emit light again [36,50]. We believe that this is a likely scenario for S1. In the case of S2, when there is no complete deactivation of the defect but only partial suppression of the radiation intensity occurs, the above mechanism is possible for a defect near S2. Then, for example, an electric field can change near S2, on the one hand, reducing the probability of the electron transition from the lower excited to the ground state and, on the other hand, increasing the probability of the transition to the metastable state. In order for the defect to be activated (turn to the bright state), it is necessary that the electron located at a electron trap (5) can be excited and get back into the CB and then return to the original defect. The probability of excitation and return for the electron apparently depends on the pump power, as can be seen from our measurements. The probability also depends on the distance between the fluorescent defect and the electron trap and the presence or absence of electron-filled neighboring traps [51]. The presence of other empty traps may lead to a decrease in the activation probability for the defect. Other mechanisms of defect activation are also possible, which have been discussed in [31,36,50].

The transition from level (3) to level (2) can also limit the total PL from the defects. However, we do not observe an obvious saturation behavior of $R_{0}\left(P\right)$ for the given emitters which can be due to the fact that $\kappa_{32}$ is close to $\Gamma_{0}$, as obtained in ref. [45]. Apparently, $\kappa_{32}$ is significantly greater than $\Gamma_{0}$; therefore, saturation is unlikely to arise at the excitation power we deal with.

### 3.3. Defects Nature

The local environment of the defect can greatly affect both the rate of emission from the defect and its radiation stability in various pumping modes. For instance, defects such as boron dangling bonds (DB) are considered as one of the examples [52,53]. In such defect, the energy and rate of the main electronic transition may depend on the local stress and the orientation of the boron atom relative to the hBN plane. According to the theoretical study [54], the height displacement of boron can lead to a decrease in excited-state decay times, which is similar to our study. In addition, it has been demonstrated that applying an electric field perpendicular to the hBN surface can change the excited-state decay time [55]. However, we also do not exclude that other defects [12,56,57], for example, carbon impurities or carbon donor-acceptor dimers, may be responsible for the emission of defects in the visible range [15,58]. Finally, the effect of local environments on the excited-state decay time can correspond to localization of the given defects near the flake surface, as observed in ref. [24].

## 4. Conclusions

Our study reveals emitters with high-intensity emission and subnanosecond excited-state lifetimes. We demonstrate that with high-power excitation, a transition rate exceeding 10 GHz with pure single-photon generation is achieved without any special resonance environment. We show that the decay times observed for the SPE in hBN are related to both the atomic structure of defects and their localization relative to other optically inactive defects. Photoswitching emission measurements reveal the electronic level configurations, in particular the presence of dark states and an intermediate excited level. The given results shed more light on the origin of quantum sources in hBN emitting at 1.9–2.2 eV with high decay rates and can pave the way for the fabrication of the SPEs with indistinguishable photons [59].

## Figures and Tables

**Table 1:** Simulated transition rates for the S1 and S2 emitters in the dark and bright states.

	$\gamma_{12}$ (GHz)	$\gamma_{21}$ (GHz)	$\kappa_{24}$ (MHz)	$\kappa_{41}$ (MHz)	$\kappa_{25}$ (MHz)	$\kappa_{51}$ (MHz)
S1 (bright state)	0.27	0.88	2.7	2.6	-	-
S1 (dark state)	0.13	0.7	16.5	3.2	2.3	1.4
S2 (bright state)	0.11	1.5	4.9	2.5	-	-
S2 (dark state)	0.93	1.7	10	0.7	-	-

## Data Availability

Not applicable.

## Abbreviations

The following abbreviations are used in this manuscript:

hBN	hexagonal boron nitride
SPE	single photon emitter
AFM	atomic force microscopy
ZPL	zero-phonon line
PL	photoluminescence
CW	continuous wave
HBT	Hanbury Brown-Twiss
IRF	instrument response function
PSB	phonon sideband
TCSPC	time-correlated single photon counting
CB	conduction band
DB	dangling bond

## Appendix D. Photon Autocorrelation Simulation

We adapted the simulation approach described in our previous study [36] with an additional optimization step to find the best fits for experimental $g^{2}\left(\tau \right)$ curves. Here, we briefly discuss the main ideas.

Consider a four-level structure (Figure 4d) to describe the PL observed from S1 and S2 emitters in the dark and bright states. According to our observations, $\kappa_{32}\gg \gamma_{21}∼\Gamma_{0}$. Hence, there is no need to consider an additional level (3) for evaluating $g^{2}\left(\tau \right)$. We introduce the following optical rate equation:

(A1) $$\dot{\rho }=R\rho ,$$

where ρ is the state populations vector, and R is the transition rates matrix given by

$$R=\left(\begin{array}{ccc} -\gamma_{12} & \gamma_{21} & \kappa_{41}\\ \gamma_{12} & -\gamma_{21}-\kappa_{24} & 0\\ 0 & \kappa_{24} & -\kappa_{41} \end{array}\right),$$

where $\gamma_{ij}$ and $\kappa_{ij}$ are the rates of radiative and non-radiative transitions from state *i* to state *j*. We can find the time evolution of the radiative state ($\rho_{2}$) to calculate $g^{\left(2\right)}\left(\tau \right)$ [38]:

$$g^{\left(2\right)}\left(\tau \right)=\frac{\rho_{2}\left(t_{2}|\rho \left(t_{1}\right)\right)}{\rho_{2}\left(t\to \infty \right)},$$

where $\tau =t_{1}-t_{2}$. To solve the differential equation (Equation (A1)), we used the ode15s function in MATLAB, fitting the solution to the experimental $g^{\left(2\right)}\left(\tau \right)$ by using Function Handle. Table 1 shows the calculated transition rates for the S1 and S2 emitters in the dark and bright states. The calculated $g^{\left(2\right)}\left(\tau \right)$ curves are demonstrated in Figure 4f. For S1 in the dark state, there is an additional metastable state (5), and the transition rates for it are set in a way similar to the (4) state.

## References

[1] Aharonovich I., Englund D., Toth M. (2016). Solid-state single-photon emitters. Nat. Photonics.

[2] Zeng H.Z.J., Ngyuen M.A.P., Ai X., Bennet A., Solnstev A.S., Laucht A., Al-Juboori A., Toth M., Mildren R.P., Malaney R. (2022). Integrated room temperature single-photon source for quantum key distribution. Opt. Lett..

[3] Zhong H.S., Wang H., Deng Y.H., Chen M.C., Peng L.C., Luo Y.H., Qin J., Wu D., Ding X., Hu Y. (2020). Quantum computational advantage using photons. Science.

[4] Wang Q., Zheng Y., Zhai C., Li X., Gong Q., Wang J. (2021). Chip-based quantum communications. J. Semicond..

[5] Arakawa Y., Holmes M.J. (2020). Progress in quantum-dot single photon sources for quantum information technologies: A broad spectrum overview. Appl. Phys. Rev..

[6] Lukishova S.G., Bissell L.J. (2019). Nanophotonic advances for room-temperature single-photon sources. Quantum Photonics: Pioneering Advances and Emerging Applications.

[7] Castelletto S., Inam F.A., Sato S.i., Boretti A. (2020). Hexagonal boron nitride: A review of the emerging material platform for single-photon sources and the spin–photon interface. Beilstein J. Nanotechnol..

[8] Vasiliev R.B., Sokolikova M., Vitukhnovskii A.G., Ambrozevich S., Selyukov A., Lebedev V.S. (2015). Optics of colloidal quantum-confined CdSe nanoscrolls. Quantum Electron..

[9] Grosso G., Moon H., Lienhard B., Ali S., Efetov D.K., Furchi M.M., Jarillo-Herrero P., Ford M.J., Aharonovich I., Englund D. (2017). Tunable and high-purity room temperature single-photon emission from atomic defects in hexagonal boron nitride. Nat. Commun..

[10] Boll M.K., Radko I.P., Huck A., Andersen U.L. (2020). Photophysics of quantum emitters in hexagonal boron-nitride nano-flakes. Opt. Express.

[11] Nikolay N., Mendelson N., Özelci E., Sontheimer B., Böhm F., Kewes G., Toth M., Aharonovich I., Benson O. (2019). Direct measurement of quantum efficiency of single-photon emitters in hexagonal boron nitride. Optica.

[12] Kubanek A. (2022). Coherent Quantum Emitters in Hexagonal Boron Nitride. Adv. Quantum Technol..

[13] Shtansky D.V., Matveev A.T., Permyakova E.S., Leybo D.V., Konopatsky A.S., Sorokin P.B. (2022). Recent Progress in Fabrication and Application of BN Nanostructures and BN-Based Nanohybrids. Nanomaterials.

[14] Chen Y., Li C., White S., Nonahal M., Xu Z.Q., Watanabe K., Taniguchi T., Toth M., Tran T.T., Aharonovich I. (2021). Generation of High-Density Quantum Emitters in High-Quality, Exfoliated Hexagonal Boron Nitride. Acs Appl. Mater. Interfaces.

[15] Mendelson N., Chugh D., Reimers J.R., Cheng T.S., Gottscholl A., Long H., Mellor C.J., Zettl A., Dyakonov V., Beton P.H. (2021). Identifying carbon as the source of visible single-photon emission from hexagonal boron nitride. Nat. Mater..

[16] Xu X., Martin Z.O., Sychev D., Lagutchev A.S., Chen Y.P., Taniguchi T., Watanabe K., Shalaev V.M., Boltasseva A. (2021). Creating quantum emitters in hexagonal boron nitride deterministically on chip-compatible substrates. Nano Lett..

[17] Gan L., Zhang D., Zhang R., Zhang Q., Sun H., Li Y., Ning C.Z. (2022). Large-Scale, High-Yield Laser Fabrication of Bright and Pure Single-Photon Emitters at Room Temperature in Hexagonal Boron Nitride. ACS Nano.

[18] Ziegler J., Klaiss R., Blaikie A., Miller D., Horowitz V.R., Alemán B.J. (2019). Deterministic quantum emitter formation in hexagonal boron nitride via controlled edge creation. Nano Lett..

[19] Glushkov E., Macha M., Rath E., Navikas V., Ronceray N., Cheon C.Y., Ahmed A., Avsar A., Watanabe K., Taniguchi T. (2022). Engineering optically active defects in hexagonal boron nitride using focused ion beam and water. ACS Nano.

[20] Kumar A., Cholsuk C., Zand A., Mishuk M.N., Matthes T., Eilenberger F., Suwanna S., Vogl T. (2022). Localized creation of yellow single photon emitting carbon complexes in hexagonal boron nitride. arXiv.

[21] Tawfik S.A., Ali S., Fronzi M., Kianinia M., Tran T.T., Stampfl C., Aharonovich I., Toth M., Ford M.J. (2017). First-principles investigation of quantum emission from hBN defects. Nanoscale.

[22] Abdi M., Chou J.P., Gali A., Plenio M.B. (2018). Color centers in hexagonal boron nitride monolayers: A group theory and ab initio analysis. ACS Photonics.

[23] Xu Z.Q., Elbadawi C., Tran T.T., Kianinia M., Li X., Liu D., Hoffman T.B., Nguyen M., Kim S., Edgar J.H. (2018). Single photon emission from plasma treated 2D hexagonal boron nitride. Nanoscale.

[24] Vogl T., Doherty M.W., Buchler B.C., Lu Y., Lam P.K. (2019). Atomic localization of quantum emitters in multilayer hexagonal boron nitride. Nanoscale.

[25] Fröch J.E., Li C., Chen Y., Toth M., Kianinia M., Kim S., Aharonovich I. (2022). Purcell Enhancement of a Cavity-Coupled Emitter in Hexagonal Boron Nitride. Small.

[26] Selyukov A., Danilkin M., Eliseev S.P., Kuznetsov A.S., Grafova V.P., Klimonsky S.O., Vainer Y.G., Vasiliev R.B., Vitukhnovsky A.G. (2020). Luminescence relaxation dynamics for planar and rolled-up CdSe nanocrystals in a photonic-crystal matrix. Quantum Electron..

[27] Vogl T., Lecamwasam R., Buchler B.C., Lu Y., Lam P.K. (2019). Compact cavity-enhanced single-photon generation with hexagonal boron nitride. ACS Photonics.

[28] Gritsienko A., Kurochkin N., Vitukhnovsky A., Selyukov A., Taydakov I., Eliseev S. (2019). Radiative characteristics of nanopatch antennas based on plasmonic nanoparticles of various geometry and tris (2, 2’-bipyridine) ruthenium (II) hexafluorophosphate. J. Phys. D Appl. Phys..

[29] Parto K., Azzam S.I., Lewis N., Patel S.D., Umezawa S., Watanabe K., Taniguchi T., Moody G. (2022). Cavity-Enhanced 2D Material Quantum Emitters Deterministically Integrated with Silicon Nitride Microresonators. arXiv.

[30] Grevtseva I.G., Ovchinnikov O.V., Smirnov M.S., Perepelitsa A.S., Chevychelova T.A., Derepko V.N., Osadchenko A.V., Selyukov A.S. (2022). The structural and luminescence properties of plexcitonic structures based on Ag 2 S/l-Cys quantum dots and Au nanorods. RSC Adv..

[31] Kianinia M., Bradac C., Sontheimer B., Wang F., Tran T.T., Nguyen M., Kim S., Xu Z.Q., Jin D., Schell A.W. (2018). All-optical control and super-resolution imaging of quantum emitters in layered materials. Nat. Commun..

[32] Noh G., Choi D., Kim J.H., Im D.G., Kim Y.H., Seo H., Lee J. (2018). Stark tuning of single-photon emitters in hexagonal boron nitride. Nano Lett..

[33] Klokov A.Y., Frolov N.Y., Sharkov A.I., Nikolaev S.N., Chernopitssky M.A., Chentsov S.I., Pugachev M.V., Duleba A.I., Shupletsov A.V., Krivobok V.S. (2022). 3D Hypersound Microscopy of van der Waals Heterostructures. Nano Lett..

[34] Pugachev M.V., Duleba A.I., Galiullin A.A., Kuntsevich A.Y. (2021). Micromask lithography for cheap and fast 2D materials microstructures fabrication. Micromachines.

[35] Li C., Xu Z.Q., Mendelson N., Kianinia M., Toth M., Aharonovich I. (2019). Purification of single-photon emission from hBN using post-processing treatments. Nanophotonics.

[36] Gritsienko A.V., Matveev A.T., Kurochkin N.S., Voskanyan G.R., Shcherbakov D.A., Shtansky D.V., Vitukhnovsky A.G. (2022). Photocontrol of Single-Photon Generation in Boron Nitride Nanoparticles: Implications for Quantum Photon Sources, Sub-Diffraction Nanoscopy, and Bioimaging. ACS Appl. Nano Mater..

[37] Gritsienko A., Kurochkin N., Lega P., Orlov A., Ilin A., Eliseev S., Vitukhnovsky A. (2021). Hybrid cube-in-cup nanoantenna: Towards ordered photonics. Nanotechnology.

[38] Fishman R.E., Patel R.N., Hopper D.A., Huang T.Y., Bassett L.C. (2021). Photon emission correlation spectroscopy as an analytical tool for quantum defects. arXiv.

[39] Kurochkin N., Savinov S., Bi D., Sychev V., Eliseev S., Gritsienko A. (2021). Characterization of Milled High-Pressure High-Temperature NV-Center Nanodiamonds for Single-Photon Source Applications. J. Russ. Laser Res..

[40] Tran T.T., Zachreson C., Berhane A.M., Bray K., Sandstrom R.G., Li L.H., Taniguchi T., Watanabe K., Aharonovich I., Toth M. (2016). Quantum emission from defects in single-crystalline hexagonal boron nitride. Phys. Rev. Appl..

[41] Tran T.T., Elbadawi C., Totonjian D., Lobo C.J., Grosso G., Moon H., Englund D.R., Ford M.J., Aharonovich I., Toth M. (2016). Robust multicolor single photon emission from point defects in hexagonal boron nitride. ACS Nano.

[42] Exarhos A.L., Hopper D.A., Grote R.R., Alkauskas A., Bassett L.C. (2017). Optical signatures of quantum emitters in suspended hexagonal boron nitride. ACS Nano.

[43] Kozawa D., Rajan A.G., Li S.X., Ichihara T., Koman V.B., Zeng Y., Kuehne M., Iyemperumal S.K., Silmore K.S., Parviz D. (2019). Observation and spectral assignment of a family of hexagonal boron nitride lattice defects. arXiv.

[44] Vogl T., Campbell G., Buchler B.C., Lu Y., Lam P.K. (2018). Fabrication and deterministic transfer of high-quality quantum emitters in hexagonal boron nitride. ACS Photonics.

[45] Patel R.N., Hopper D.A., Gusdorff J.A., Turiansky M.E., Huang T.Y., Fishman R.E.K., Porat B., Van de Walle C.G., Bassett L.C. (2022). Probing the Optical Dynamics of Quantum Emitters in Hexagonal Boron Nitride. PRX Quantum.

[46] Feldman M.A., Puretzky A., Lindsay L., Tucker E., Briggs D.P., Evans P.G., Haglund R.F., Lawrie B.J. (2019). Phonon-induced multicolor correlations in hBN single-photon emitters. Phys. Rev. B.

[47] Feldman M.A., Marvinney C.E., Puretzky A.A., Lawrie B.J. (2021). Evidence of photochromism in a hexagonal boron nitride single-photon emitter. Optica.

[48] Jungwirth N.R., Fuchs G.D. (2017). Optical absorption and emission mechanisms of single defects in hexagonal boron nitride. Phys. Rev. Lett..

[49] White S.J., Yang T., Dontschuk N., Li C., Xu Z.Q., Kianinia M., Stacey A., Toth M., Aharonovich I. (2022). Electrical control of quantum emitters in a Van der Waals heterostructure. Light. Sci. Appl..

[50] Khatri P., Ramsay A.J., Malein R.N.E., Chong H.M., Luxmoore I.J. (2020). Optical gating of photoluminescence from color centers in hexagonal boron nitride. Nano Lett..

[51] Katsaba A., Fedyanin V., Ambrozevich S., Vitukhnovsky A., Lobanov A., Selyukov A., Vasiliev R., Samatov I., Brunkov P. (2013). Characterization of defects in colloidal CdSe nanocrystals by the modified thermostimulated luminescence technique. Semiconductors.

[52] Turiansky M.E., Alkauskas A., Bassett L.C., Van de Walle C.G. (2019). Dangling bonds in hexagonal boron nitride as single-photon emitters. Phys. Rev. Lett..

[53] Turiansky M.E., Van de Walle C.G. (2021). Boron dangling bonds in a monolayer of hexagonal boron nitride. J. Appl. Phys..

[54] Turiansky M.E., Van de Walle C.G. (2021). Impact of dangling bonds on properties of h-BN. 2D Mater..

[55] Scavuzzo A., Mangel S., Park J.H., Lee S., Loc Duong D., Strelow C., Mews A., Burghard M., Kern K. (2019). Electrically tunable quantum emitters in an ultrathin graphene–hexagonal boron nitride van der Waals heterostructure. Appl. Phys. Lett..

[56] McDougall N.L., Partridge J.G., Nicholls R.J., Russo S.P., McCulloch D.G. (2017). Influence of point defects on the near edge structure of hexagonal boron nitride. Phys. Rev. B.

[57] Chen Y., Quek S.Y. (2021). Photophysical Characteristics of Boron Vacancy-Derived Defect Centers in Hexagonal Boron Nitride. J. Phys. Chem. C.

[58] Li K., Smart T., Ping Y. (2021). *C*_2_*C*_*N*_ as a 2 eV Single-Photon Emitter Candidate in Hexagonal Boron Nitride. arXiv.

[59] Wein S., Lauk N., Ghobadi R., Simon C. (2018). Feasibility of efficient room-temperature solid-state sources of indistinguishable single photons using ultrasmall mode volume cavities. Phys. Rev. B.

